# Effects of Maternal Diabetes and Diet on Gene Expression in the Murine Placenta

**DOI:** 10.3390/genes13010130

**Published:** 2022-01-12

**Authors:** Claudia Kappen, Claudia Kruger, J. Michael Salbaum

**Affiliations:** 1 Department of Developmental Biology, Pennington Biomedical Research Center—Louisiana State University System, 6400 Perkins Road, Baton Rouge, LA 70808, USA; claudia.kruger@pbrc.edu; 2 Department of Regulation of Gene Expression, Pennington Biomedical Research Center—Louisiana State University System, 6400 Perkins Road, Baton Rouge, LA 70808, USA; michael.salbaum@pbrc.edu

**Keywords:** diabetic pregnancy, placenta formation, trophoblast migration, intrauterine growth restriction

## Abstract

Adverse exposures during pregnancy have been shown to contribute to susceptibility for chronic diseases in offspring. Maternal diabetes during pregnancy is associated with higher risk of pregnancy complications, structural birth defects, and cardiometabolic health impairments later in life. We showed previously in a mouse model that the placenta is smaller in diabetic pregnancies, with reduced size of the junctional zone and labyrinth. In addition, cell migration is impaired, resulting in ectopic accumulation of spongiotrophoblasts within the labyrinth. The present study had the goal to identify the mechanisms underlying the growth defects and trophoblast migration abnormalities. Based upon gene expression assays of 47 candidate genes, we were able to attribute the reduced growth of diabetic placenta to alterations in the Insulin growth factor and Serotonin signaling pathways, and provide evidence for Prostaglandin signaling deficiencies as the possible cause for abnormal trophoblast migration. Furthermore, our results reinforce the notion that the exposure to maternal diabetes has particularly pronounced effects on gene expression at midgestation time points. An implication of these findings is that mechanisms underlying developmental programming act early in pregnancy, during placenta morphogenesis, and before the conceptus switches from histiotrophic to hemotrophic nutrition.

## 1. Introduction

Diabetes during pregnancy is a known risk factor for congenital defects and pregnancy complications, such as spontaneous abortions and preeclampsia [1,2,3,4]. Progeny from diabetic pregnancies also have a higher risk for metabolic disease later in life [5,6,7], which may be mediated by lower or higher than average weight at birth, by fetal hyperinsulinemia [8], or by as yet unidentified mechanisms of developmental programming [9,10].

In a mouse model of type I diabetes, we previously showed that fetal growth is reduced, noticeable after gestational day 15.5, and that the growth reduction was more pronounced when the dam was fed a diet with higher fat and lower protein content compared to chow [11]. While placenta size was not altered by chow diet, placental growth was impaired during diabetic pregnancy with the higher fat content diet [12]. In particular, the spongiotrophoblast layer and the labyrinth area were significantly reduced in thickness by gestational days 15.5 and 18.5, respectively [13]. The present study was therefore designed to investigate—in females fed the higher fat content diet—the effects of diet on the expression of a number of genes implicated in placenta growth and fetal growth, including Insulin-like growth factors (Igfs), and their receptor and binding protein genes.

In addition, even with normal diet, the contribution of spongiotrophoblast cells to the junctional zone of the placenta was decreased in diabetic pregnancies, and cells expressing the spongiotrophoblast marker Trophoblast-specific protein alpha (*TpbpA*) ectopically accumulated in the labyrinth [13]. This suggested to us that cell differentiation and cell migration is altered in the placenta under conditions of maternal hyperglycemia, and, potentially, affected by maternal diet. As migration and invasion of the maternal decidua by trophoblasts requires remodeling of the extracellular matrix, the present study also examines whether the impaired trophoblast migration in diabetic placentae could be mediated by altered expression of metalloproteases or their inhibitors. Furthermore, we wanted to assess to what extent maternal diet affects the expression of these genes in the placenta. A crucial link between metalloproteinase expression and Insulin-like growth factor signaling are the pregnancy-associated plasma proteins metalloproteases which are involved in the release of bioactive Igf from Igf-binding proteins.

Because metalloproteinase action can be a sign of inflammation, we also investigated the expression of genes involved in altered immune responses and inflammation, including genes in the prostaglandin pathway. Prostaglandins are well-known mediators of inflammation, and play a critical role in the timing of implantation and parturition [14]. It is known that prostaglandin signaling affects trophoblast invasion [15,16], and, therefore, we reasoned that altered prostaglandin signaling might contribute to the impaired placenta development in diabetic pregnancies. The selection of genes to be investigated in this study was guided by our earlier gene expression profiling results that had demonstrated their expression in normal and diabetic placentae [13].

## 2. Materials and Methods

Diabetes was induced in FVB mice by injection of Streptozotocin, as described previously [11,12]. The blood glucose levels and morphometric parameters of pregnant dams have been published [11,12]. Archived placenta samples were used in the present study that had been collected and processed for RNA extraction as published, and quantitative PCR was performed exactly as before [11,12,17,18].

Diets consisted of chow diet (Labdiet Purina 5001) until FVB females were randomly assigned at eight weeks of age to either continue feeding on chow, or breeder diet (Purina 5015), which is formulated specifically to support pregnancy and lactation. The compositions of the diets are described in the manufacturer’s datasheets (see Supplemental Files) and differ in protein and fat content, with minor variation in carbohydrate content. Importantly, both diets are nutritionally complete for mice in normal metabolic conditions. Females consumed the respective diet for at least four weeks before mating.

Primers for real-time quantitative PCR amplification were designed following the criteria described in Kruger et al. (2006) [17]. The gene encoding DNA Polymerase epsilon 4 was used as the reference gene for normalization. Where possible, primers were positioned so that the resulting amplicon would span an exon–exon junction so that amplicons would be derived only from cDNA templates. Sequences of primers and their coordinates are listed in Table 1. Primers for genes assayed in the current study but not included on this list have been published previously [12].

Our method for quantitation of gene expression levels was applied exactly as in previous studies [11,12,17,18]. Measurements on each sample were done in triplicates and obtained values were averaged. Amplification efficiencies were calculated, and statistical evaluation was performed on ∆C_T_ values. Experimental groups consisted of six placentae, each from a separate diabetic or control pregnancy, respectively [12,18]. Figures show inverse ∆C_T_ to depict the highest expression as the highest visual points on the graphs.

Two-tailed T-tests—with assumption of unequal variance—were performed to evaluate significance of differences in gene expression levels between groups; a *p*-value of <0.05 was considered significant.

In situ hybridizations were performed on frozen sections as described [13], with most probes obtained from Open Biosystems/Thermo Fisher.

## 3. Results and Discussion

**Figure 1:**
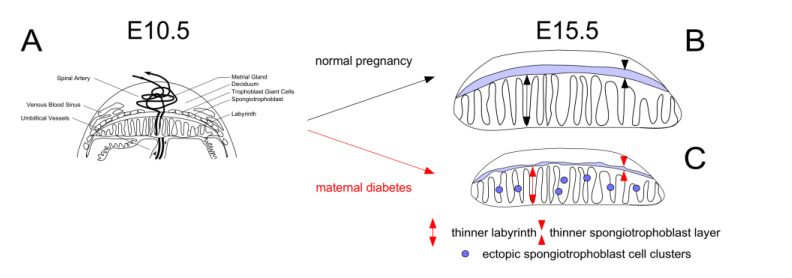
**Overview of placental anomalies in mouse diabetic pregnancies.Panel A**: Schematic depiction of the placenta at E10.5. Compared to normal pregnancies (**Panel B**), the placenta remains smaller in diabetic pregnancies (**Panel C**), with reduced thickness of labyrinth and junctional zone, and ectopic accumulation of spongiotrophoblasts in the labyrinth, due to impaired cell migration. Placenta weights were lowest in diabetic pregnancies where the dam was fed a diet (Breeder diet) that is optimized to promote pregnancy and lactation under normal conditions.

Our prior studies provided evidence that in diabetic mouse pregnancies, placentae are smaller and that their junctional zones are thinner, when compared to placentae from metabolically normal pregnancies [13]. We also showed that maternal diabetes affects the migration of spongiotrophoblast cells, some of which form ectopic cell clusters in the labyrinth instead of contributing to the junctional layer (Figure 1).

The reductions in placental size are more pronounced in diabetic pregnancies when dams are fed breeder diet compared to chow [12]. Taken together, these observations prompted us to investigate the potential underlying mechanisms, focusing on genes known to be involved in embryonic and placental growth, trophoblast invasion, cell migration, and inflammatory processes. Because we showed earlier that the growth differences and placental cellular abnormalities in late pregnancy are preceded by alterations in gene expression at midgestation [12,13], we assayed placental mRNA levels at various gestational time points.

Expression of 47 genes was measured in placenta samples from normal and diabetic pregnancies, where dams had been fed either chow or breeder diet for four weeks prior to mating. In all graphs presented here, chow-fed experimental groups are represented by open symbols and dashed lines, while breeder diet-fed groups are depicted by closed symbols and solid lines; the diabetic condition is always marked in red.

**Figure 2:**
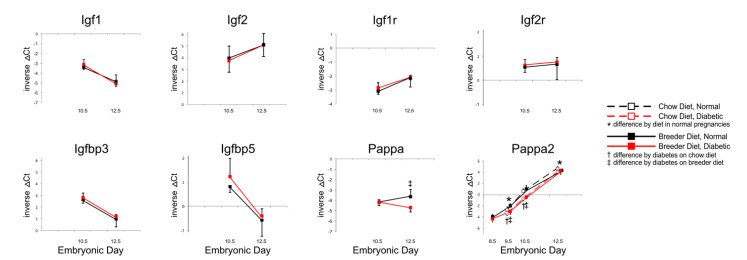
**Expression of Igfs, their receptors, Igf-binding proteins and their proteases.** Quantitative PCR measurements were performed at various time points, in dams that were either fed chow or breeder diet, and became diabetic in the week before, or were normoglycemic at the time of mating.

The Insulin growth factor signaling system has long been known to regulate embryonic growth and placental size in the mouse [19]. However, as Figure 2 shows, no significant differences were detected in the expression levels of genes encoding Igf 1, Igf2, or their respective receptors, nor the two Igf-binding proteins known to be expressed in mouse placenta. *Pregnancy-associated protein A (Pappa)* transcript levels were lower in diabetic placentae compared to controls, but only at E12.5; *Pappa2* levels were also lower with maternal diabetes, from E9.5 on, with both diets.

These results suggest that reduced expression of Pappa proteins could play a role in the growth deficiency of placentae under diabetic conditions, as Pappa metalloproteases have Igf-binding proteins as their main targets [20,21], and reduced proteolytic activity would result in reduced Igf availability [22]. Consistent with this proposition is the finding that targeted disruption of the *Pappa* gene in mice causes intrauterine growth restriction [23]. In the case of Pappa2 deficiency, growth retardation was only evident postnatally [24], but this does not exclude the possibility that Pappa2 reduction in diabetic pregnancies could influence postnatal growth trajectories, as offspring from mouse diabetic pregnancies are born smaller [11], and intrauterine growth-restricted progeny can experience substantial catch-up growth [25,26].

We then investigated several genes that are known to be required for embryonic development from defects reported with targeted disruption, such as for example embryonic lethality in mice with targeted disruption of *Proprotein convertase subtilisin/kexin type 5 (Pcsk5)* [27] and *Bone morphogenetic protein 2 (Bmp2)* [28], multiple defects in mutants for *endoplasmatic reticulum metallopeptidase Ermp1* [29], *Calcitonin related peptide beta (Calcb)* [30], *Mmp14* [31], and overall growth delay as in mutants for the *Tgf-beta induced Tgfbi* gene [32]. Atonal bHLH transcription factor 8 (Atoh8/Math6)-deficient mice exhibit embryonic lethality, due to placental defects [33], which are also suspected but as of yet uncharacterized in many embryonic and early postnatal lethal mouse mutants [34]. In addition, we assayed genes with documented expression in specific placental cell types, such as *Ascl2* in the labyrinth and spongiotrophoblasts [35], the *Slc6a4 serotonin transporter* in trophoblast giant cells [36], *TpbpA* in spongiotrophoblasts [37], *Cck* in cells surrounding the spiral arteries [13], and the prolactin *Prl5a1*, which is expressed in spongiotrophoblasts [38].

**Figure 3:**
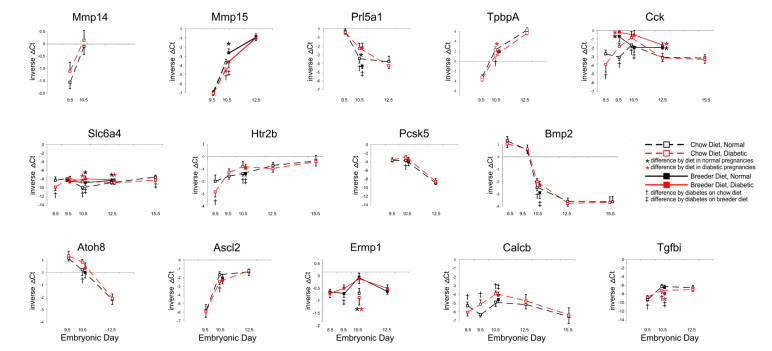
**Expression of genes with roles in embryonic and placental development.** Quantitative PCR measurements were performed at various time points, in dams that were either fed chow or breeder diet, and became diabetic in the week before, or were normoglycemic at the time of mating.

As shown in Figure 3, many of these genes exhibited significantly different expression levels in placentae from diabetic pregnancies at midgestation, i.e., E10.5, while differences at later stages were detected only for *Cck* and *Slc6a4*. Higher expression of *Cck* was observed in breeder-diet-fed dams, but differences between normal and diabetic placentae were no longer present after E10.5, after which diet appeared to be the main driver. *Slc6a4* displayed a similar pattern, at a moderate level of expression up until E12.5, but its expression was significantly lower in diabetic placentae by E15.5. Although transcripts for the *Serotonin receptor Htr2b* only differed at E10.5, the decreased expression of the *Serotonin transporter Slc6a4* suggests that placental serotonin signaling could be impaired at later stages of gestation. Since Serotonin acts as a growth factor during development [39,40,41,42], reduced availability in the second half of pregnancy may contribute to the smaller placenta size in diabetic pregnancies. Furthermore, it was recently proposed that Serotonin may control placental nutrient uptake [43].

However, from some of the temporal trajectories it is not intuitively obvious how changes in expression of these genes could explain the reduced growth of the placenta after E12.5, particularly in diabetic breeder-diet-fed dams. It cannot be excluded that the observed changes at midgestation (E10.5, or even earlier) could subsequently influence signaling pathways that were not included in our investigations. It is important to note here that technical factors are unlikely to account for the large number of genes with differences at E10.5, as both higher and lower transcript levels of individual genes were measured in diabetic placentae compared to normal. Furthermore, gene expression in distinct cell types may respond uniquely to diet and maternal diabetes. Likewise, the timing of responses to metabolic conditions may also be cell-type-specific. As embryonic blood circulation is believed not to become fully established until E10 [44], the transcriptional responses of placental genes at E10.5 could also reflect the newly developed capacity for, and the consequences of, oxidative metabolism in the embryo, after the switch from histotrophic to hemotrophic nutrition. Furthermore, we have previously shown that the responses of placental genes to influences of diet and maternal metabolic disease follow diverse patterns—with reactivity to either one of these conditions or both conditions combined—that produce additive as well as antipodal effects on expression levels [12]. Taken together, these considerations suggest that likely multiple pathways, possibly in different phases of gestation, contribute to reduced placenta size in diabetic pregnancies.

Signaling pathways that are known or could be suspected to be involved in decidualization and in trophoblast invasion of the decidua, or in trophoblast migration were also assayed. Prostaglandin signaling has been implicated in these processes previously [45,46,47,48,49]. Prostanoid signaling molecules are produced from the polyunsaturated fatty acid Arachidonic acid, which is released from phospholipids by phospholipases, such as Lipoprotein lipase (Lpl) and the Pla2 enzymes. Various enzymatic steps can then produce prostanoids with pro- or anti-inflammatory properties. Potential aberrations in these processes in diabetic pregnancies are suggested by prior evidence that prostanoid production is altered in diabetic rat placenta [50], and that supplementation of Arachidonic acid and/or Prostaglandins can reduce the incidence of developmental defects in rodent diabetic pregnancies [51,52,53,54,55]. Hydroxy-prostaglandindehydrogenase (Hpgd) and Carbonylreductase (Cbr1) are two enzymes controlling prostaglandin availability, as does the Slco2a1 prostaglandin transporter [14], and Glutathione peroxidase 4 (Gpx4) is critically required to eliminate lipid radicals that form when unsaturated fatty acids are involved in lipid peroxidation under conditions of oxidative stress [56]. Elevated oxidative stress has been documented in placentae from rodent diabetic pregnancies [50].

**Figure 4:**
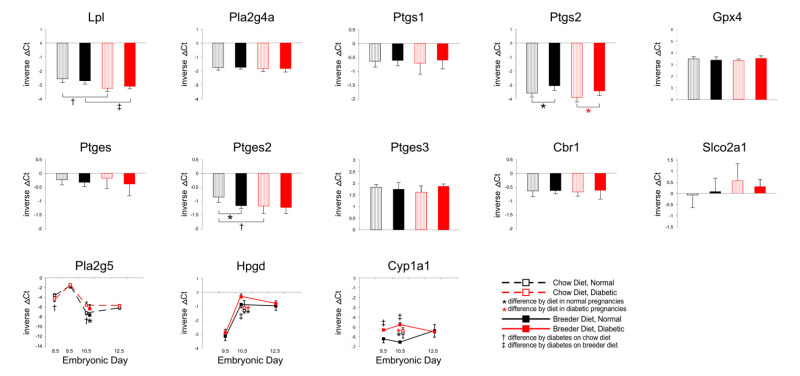
**Expression of genes involved in prostaglandin synthesis and metabolism.** Quantitative PCR measurements were performed at various time points, in dams that were either fed chow or breeder diet, and became diabetic in the week before, or were normoglycemic at the time of mating. Data in bar diagrams were obtained from placentae isolated at E10.5.

Figure 4 displays gene expression measurements for several of the enzymes involved in the Arachidonic acid–Prostaglandin pathway. Intriguingly, *Lpl* expression was found to be lower in diabetic pregnancies, but there were no differences in *Phospholipase 2g4a* (Pla2g4) nor *Prostaglandin endoperoxide synthase (Ptsg1/Cox-1)* expression. While not altered by maternal diabetes, *Ptgs2 (Cox-2)* levels were elevated when the dam was fed breeder diet, possibly indicative of increased prostanoid production under these dietary conditions. Unaltered *Gpx4* expression, at least at the E10.5 time point measured, did not provide evidence this would be associated with increased lipid peroxidation. *Phospholipase 2g5* transcript levels were elevated by diet, and by maternal diabetes, too, but only at E10.5. Of the Prostaglandin E synthesizing enzymes, only *Ptges2* levels were changed, reduced by diet, and reduced by maternal diabetes. While *Cbr1* levels did not change, the apparent increase of *Slco2a1 prostaglandin transporter* expression in diabetic conditions was not significant, due to large variations between individuals within each experimental group. Intriguingly, *Hpgd* levels were higher with breeder diet feeding, and in diabetic conditions. This was also the case for expression of *Cyp1a1*, which can metabolize a wide variety of polyunsaturated lipids and their derivatives, including Arachidonic acid [57]. Taken together, these results would be consistent with reduced synthesis and increased elimination of Arachidonic acid and Prostaglandin E2 in diabetic conditions.

As prostanoids can act in pro- and anti-inflammatory roles, we also surveyed genes that are known to be involved in inflammation. We had showed previously that *Ankrd2* [13], which modulates NF-κB inflammatory responses [58], is specifically expressed in the placental labyrinth. Here, we focused on Metalloproteases 13, 1a, and 8, and protease inhibitors Kazal-type serine peptidase inhibitor domain 1 (Kazald1) which also contains an Igf-binding domain, the Kazal-type serine peptidase inhibitor Spink8, Serine protease inhibitor 16 (Spi16) and Thrombospondin type 1 domain containing 4 (Thsd4). Involved in turnover of extracellular matrix [59], this group of molecules contribute to the regulation of cell adhesion and migration [60,61,62]. Additional candidate genes encode Rnase2a, which attracts macrophages [63], Pore-forming protein-like (Pfpl), which is proposed to enhance trophoblast invasiveness [64], and Cysteine-rich C-terminal protein 1 (Crct1), which has been implicated in apoptosis [65].

**Figure 5:**
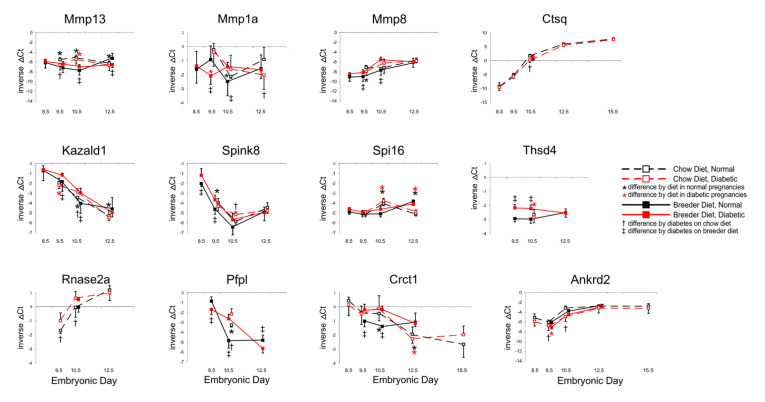
**Expression of genes involved in inflammation, cell adhesion and migration.** Quantitative PCR measurements were performed at various time points, in dams that were either fed chow or breeder diet, and became diabetic in the week before, or were normoglycemic at the time of mating.

Figure 5 demonstrates that these genes were all affected by maternal diet and diabetes. *Mmp13* levels were lower under diabetic conditions in chow-fed dams, but higher when breeder diet was consumed; by E12.5, diabetes was associated with lower-than-normal expression, particularly in dams fed breeder diet. An effect of diet on *Mmp1a* transcripts was only evident at E10.5, with antipodal effects of diabetes at various time points. In contrast, consistently higher expression of *Mmp8* was seen under diabetic conditions at E9.5 and E10.5. Lower *Cathepsin Q* levels were detected at E10.5 in chow-fed diabetic dams, but not at other time points or metabolic conditions. Maternal diabetes and dietary regimen affected *Kazald1* expression at various time points with both dietary regimen, but as for *Mmp13* and *Mmp1a*, trajectories changed over time. Likewise, expression of *Spink8* reacted to diabetes and diet, whereas *Spi16* was influenced only by diet at E10.5 and E12.5, in both metabolic conditions. *Thsd4* levels were higher in diabetic than normal placentae, and normalized by E12.5. *Rnase2* and *Pfpl* expression differed by metabolic condition at E9.5 and E10.5, and at E10.5 and E12.5, respectively. *Crct1* transcript levels were higher in diabetic conditions with both diets, and, again, the major differences were seen at E9.5 and E10.5, after which diet had a greater influence at E12.5. *Ankrd2* exhibited lower expression in diabetic placentae of chow-fed animals; breeder diet only had an effect at E9.5. Overall, where expression levels showed the greatest differences, the diabetic condition was typically associated with increased gene expression, although this was reversed for *Ctsq* and *Ankrd2*, which displayed reduced expression in diabetic placentae. In addition, the most profound differences were again evident by E10.5, further highlighting that placental gene expression this stage appears to be particularly sensitive to dietary and metabolic conditions.

The complexity of gene-specific temporal trajectories, and of distinct responses to maternal diabetes and diet at different time points, raises the possibility that the underlying regulatory mechanisms could be highly specific to particular cell types in the placenta. The murine placenta is now known to harbor over 20 cell identities that are distinguishable on the basis of single-cell-based expression profiling [66,67]. Yet, the data currently available from such efforts are insufficient to allow unequivocal attribution of cell-type-specific gene expression (Salbaum and Kappen, unpublished observations). Based on conventional in situ hybridization methods, we previously demonstrated regionally restricted expression in the placenta for several genes in the diabetic pregnancy model, such as for *Ascl2* and *Ankrd2* in the labyrinth, *Slc6a4* in giant cells, and *Cyp1a1*, *Calcb* and *Cck* in the decidual region of the placenta [13]; these studies also uncovered expression of *Mmp9* in trophoblast giant cells, *Thsd4* in giant cells, the labyrinth and decidua, and *Sfrp5* and *Rik9130008F23* exclusively in the decidual region. In the present study, we performed in situ hybridizations at E10.5 for 14 additional genes.

**Figure 6:**
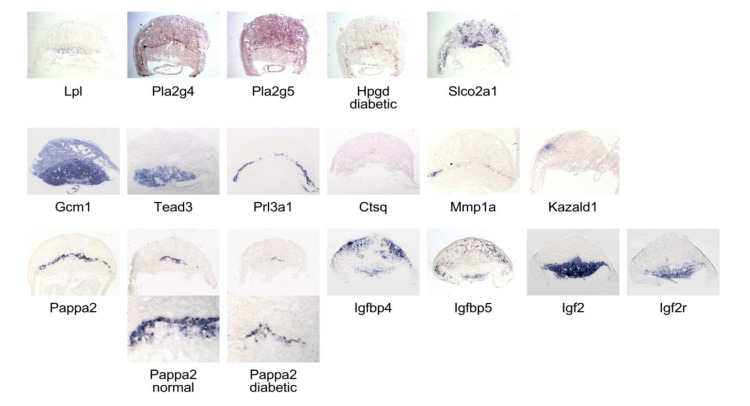
**In situ hybridization reveals region- and cell-type-specific gene expression in normal and diabetic placentae.** Sections were produced from OCT-embedded frozen tissue from normoglycemic dams, except where indicated that the sample came from a diabetic female.

Figure 6 reveals that *Lpl* expression is strongest in the labyrinthine region, while *Pla2g4* and *Pla2g5* are predominantly expressed in the decidual region, with *Pla2g4* also present in a layer of cells in the junctional zone in apposition to the labyrinth. The junctional zone is where *Hpgd* transcripts are also found, although positive cells appear in the decidual area in diabetic placentae, too. Signal for the prostaglandin transporter *Slco2a1*, which facilitates prostaglandin degradation, is detected throughout the decidual region, and also in the junctional zone, but entirely absent from the labyrinth. These patterns are consistent with the interpretation that arachidonic acid metabolites may be preferentially generated in the maternal part of the placenta, and that prostaglandin availability may also be regulated there, as well as in the junctional zone. Taken together with our quantitative assays indicating potentially elevated elimination of prostanoids, these results suggest that the reported deficiencies of arachidonic acid and prostaglandin E2 in diabetic pregnancies [68] could have their origin in the decidua and junctional zone, and that embryonic defects arising from deficiency of AA and PGE2, or both, are the result of perturbed paracrine mechanisms in decidua and placenta. Furthermore, as reduced availability of Prostaglandin E2 has been shown to impair migration of extravillous human first-trimester trophoblast cells [69], this would provide a mechanistic explanation for our earlier observations of reduced migration of spongiotrophoblast precursors to their destination in the junctional zone and their ectopic accumulation in the labyrinth.

The labyrinth exhibits strong expression of the transcription factor *Glial cells missing 1 (Gcm1*), as reported previously [70] and of *Transcription enhancer factor TEAD3* [71]. *Crct1* and *Pfpl* transcripts were detected only in very few cells along the maternal-embryonic interface (not shown). Trophoblast giant cells express *Prolactin Prl3d1* [72], and some of them display rather faint signals for *Cathepsin Q* mRNA. *Mmp1a* expression is also seen in the junctional zone, but protease inhibitor *Kazald1* is only expressed in the decidual region, and at very low levels in labyrinth.

The junctional zone is the only area displaying strong signals for *Pappa2*, which were weaker and present in fewer cells in diabetic placenta at E10.5. This indicates that fewer spongiotrophoblasts were present already at this time point, foreshadowing the decreased thickness of the junctional zone we previously documented at E15.5 and E18.5 in diabetic placentae. The Igf binding protein transcripts *Igfbp4* and *Igfbp5* are expressed associated with maternal vessels in the decidual region, and also in the lower labyrinth. The labyrinth and spongiotrophoblasts strongly express *Igf2*, consistent with prior reports [73]; *Igf2* receptor is also expressed in those areas. These results show that Igf-binding proteins are present in both maternal and embryonic compartments of the placenta, whereas *Pappa2*, which also controls Igf availability, is exclusive to the junctional zone. Relevant to diabetic pregnancies, reduced availability of Igf2, associated with reduced *Pappa2* expression (also see Figure 1) is predicted to result in smaller placentae, and also reduced embryo growth [74,75]. Lower concentrations of free Igf2 have been linked to decreased placental nutrient transport [73,76], which, subsequently, may contribute to cause the fetal growth reduction we documented previously. Thus, our identification of *Pappa2* as a gene whose expression is decreased by maternal diabetes in the placenta implicates impaired Igf2 signaling as the underlying mechanism for intrauterine growth restriction in type I diabetic mouse pregnancies.

In summary, this study investigated potential candidate genes for growth and cellular abnormalities in placentae of mice with type I diabetes during pregnancy. Our results allow us to attribute the growth deficiencies of placentae and embryos/fetuses in those pregnancies to impaired Igf2 signaling, with possible contribution of impaired Serotonin signaling. Furthermore, we provide evidence to link impaired cell migration to abnormal prostaglandin signaling. Notwithstanding the complex responsiveness of individual genes to maternal diabetes, maternal diet, or both, an overarching theme emerging from our studies is that gene expression in the midgestation placenta (E10.5) is particularly sensitive to metabolic and nutritional perturbations, possibly related to the switch from histiotrophic to hemotrophic nutrition. The molecular mechanisms by which maternal diabetes changes transcription of placental genes to cause intrauterine restriction of placental and fetal growth in the mouse, remain to be characterized.

Finally, potential implications for human pregnancies affected by type I diabetes deserve consideration: despite some structural and cellular distinctions, the process of placenta formation is highly similar between mouse and human, employing the same transcriptional networks and molecular pathways [77]. Under conditions of type I diabetes, the elevated risk for birth defects is also shared between both species [3]. However, two major differences exist between mouse and human type I diabetic pregnancies: (i) blood glucose levels in such pregnancies are very high in mouse models [11,12], but controllable to considerably lower levels in humans [78]. (ii) The longer pregnancy in humans is associated with a longer period of fetal growth in utero, while the mouse is born comparatively immaturely [79], limiting direct comparisons of gene expression data to early stages of pregnancy. Indeed, our results highlight and are consistent with prior reports that sensitivity of placental gene expression to perturbations is particularly pronounced during early stages of placenta development [77]. implicating potential vulnerabilities at comparable stages in human pregnancies.

## Figures and Tables

**Table 1:** **PCR primers employed in this study and their amplification efficiencies.** Gene symbols follow the Mouse Genome Informatics (http://www.informatics.jax.org accessed on 31 October 2021) nomenclature: Ascl2: Achaete-scute family bHLH transcription factor 2; Bmp2: Bone morphogenetic protein 2; Calcb: Calcitonin-related polypeptide, beta; Cbr1: Carbonyl reductase 1; Cck: Cholecystokinin; Ctsq: Cathepsin Q; Gpx4: Glutathione peroxidase 4; Htr2b: 5-Hydroxytryptamine (serotonin) receptor 2B; Igf1: Insulin-like growth factor 1; Igf1r: Insulin-like growth factor 1 receptor; Igf2: Insulin-like growth factor 2; Igf2r: Insulin-like growth factor 2 receptor; Igfbp3: Insulin-like growth factor binding protein 3; Igfbp5: Insulin-like growth factor binding protein 5; Kazald1: Kazal-type serine peptidase inhibitor domain 1; Klhl5: Kelch-like 5; Klk8: Kallikrein related-peptidase 8; Mmp1a: Matrix metallopeptidase 1a (interstitial collagenase); Mmp13: Matrix metallopeptidase 13; Mmp14: Matrix metallopeptidase 14 (membrane-inserted); Mmp15: Matrix metallopeptidase 15; Mmp2: Matrix metallopeptidase 2: Mmp3: Matrix metallopeptidase 3; Mmp8: Matrix metallopeptidase 8; Mmp9: Matrix metallopeptidase 9; Pappa: Pregnancy-associated plasma protein A; Pappa2: Pappalysin 2; Pla2g4a: Phospholipase A2, group IVA (cytosolic, calcium-dependent); Pla2g5: Phospholipase A2, group V; Ptges: Prostaglandin E synthase; Ptges3: Prostaglandin E synthase 3; Ptgs1: Prostaglandin-endoperoxide synthase 1; Rbbp9: Retinoblastoma binding protein 9, serine hydrolase; Rnase2a: Ribonuclease, RNase A family, 2A (liver, eosinophil-derived neurotoxin); Sfrp5: Secreted frizzled-related sequence protein 5; Slco2a1: Solute carrier organic anion transporter family, member 2a1; Spi16: Serine protease inhibitor 16; Spink8: Serine peptidase inhibitor, Kazal type 8.

Gene Symbol	Forward Primer—Sequence	Position	Reverse Primer—Sequence	Position	Exon–Exon Boundary	Amplification Efficiency
Ascl2	GCTTGACTTTTCCAGTTGGTTAGG	1024–1047	TCTTGGGCTAGAAGCAGGTAGGT	1124–1102	yes	1.84
Bmp2	CGAAGAAGCCGTGGAGGAA	1539–1557	GGGACAGAACTTAAATTGAAGAAGAAG	1616–1590	yes	1.86
Calcb	TGGAACAGGAGGAGCAAGAGA	290–310	GGTGGCAGTGTTGCAGGAT	360–342	yes	1.85
Cbr1	CGAAGCGAGACCATCACAGA	604–623	CGCATGGACTCCTTTCTTTGTA	684–663	no	1.94
Cck	ATCCAGCAGGTCCGCAAA	263–280	TCCAGGCTCTGCAGGTTCTTA	327–307	yes	1.93
Ctsq	TGGCTATGGTTATGTGGGAAATG	921–943	CATCGTCTACCCCAGCTGTTC	995–975	yes	1.87
Gpx4	AGGCAGGAGCCAGGAAGTAATC	499–520	CACAGATCTTGCTGTACATGTCAAA	583–559	yes	1.88
Htr2b	CGGAAGAGCAGGTCAGCTACA	1741–1761	CGTGCGTCCTGCTCATCAC	1810–1792	yes	1.90
Igf1	ATGAGTCTGCTCCACTGAAAATGTA	115–139	AAGGATTTTTTTACCCCAGGAAA	186–164	no	1.85
Igf1r	GCTTCTGTGAACCCCGAGTATTT	3525–3547	TGGTGATCTTCTCTCGAGCTACCT	3606–3583	yes	1.93
Igf2	TCTCGAGGCACCCTAAATTACC	3534–3555	TCAGCAAATGCCCCTGAAAG	3628–3609	no	1.96
Igf2r	TGCAACCCTCTGCCTTATATCC	3483–3504	TGAGAGACCCATTTCCAGTAGCT	3612–3590	yes	1.94
Igfbp3	GCGTCCACATCCCAAACTGT	837–856	CTGCCCATACTTGTCCACACA	946–926	yes	1.97
Igfbp5	GCCCCGTGCTGTGTACCT	1353–1370	GGCAGCTTCATTCCGTACTTG	1478–1458	yes	1.97
Kazald1	CCCATGGCCTCGATTGAGT	732–750	CCCCTAAACTGCACAGAGATATGA	817–794	yes	1.96
Klhl5	AGGCGGAAGGATCTCAGTAGACT	1217–1239	CCCGGAAAAGTGCATTGTTT	1313–1294	yes	1.88
Klk8	GCCCACTGCAAAAAACAGAAG	700–720	CCTGCTCCGGCTGATCTCT	775–757	yes	1.93
Mmp1a	AGGCAGGTTCTACATTCGGGTAA	941–963	TGGCCAGAGAATACCTATTAAATTGA	1013–988	yes	1.95
Mmp13	AATCTATGATGGCACTGCTGACAT	478–501	GTTTGGTCCAGGAGGAAAAGC	595–575	yes	1.84
Mmp14	TCGTGTTGCCTGATGACGAT	1069–1088	TTTGGGCTTATCTGGGACAGA	1193–1173	yes	1.91
Mmp15	ATGCAGCCTACACCTACTTCTACAAG	2085–2110	CCATGAAGTCCCGCAGGAT	2192–2174	yes	1.89
Mmp2	CTGAGCTCCCAGAAAAGATTGAC	1844–1866	CATTCCCTGCGAAGAACACA	1918–1899	yes	1.92
Mmp3	GGAGGTTTGATGAGAAGAAACAATC	1308–1332	GTAGAGAAACCCAAATGCTTCAAAGA	1426–1401	yes	1.84
Mmp8	GCACACCCAAAGCCTGTGA	901-919	GAGGATGCCGTCTCCAGAAG	1005–986	yes	1.91
Mmp9	CCAAGGGTACAGCCTGTTCCT	1195–1215	GCACGCTGGAATGATCTAAGC	1268–1248	yes	1.88
Pappa	CAGATACAGCGGGACGATGA	4104–4123	TCGGTACATGTCACTGTGATGCT	4171–4149	yes	1.94
Pappa2	CCAGGGCCCTCCACAGA	4414–4430	AGGCTGCCATGCTACTATTGAAG	4549–4527	no	1.93
Pla2g4a	CAGCAAAGCACATCGTGAGTAAT	1440–1462	CGGTGCCTTTGGGTCCTT	1508–1491	yes	1.90
Pla2g5	CCCAAGGATGGCACTGATTG	460–479	TCCGAATGGCACAGTCTTTTT	541–521	yes	1.87
Ptges	CAGATGAGGCTGCGGAAGA	188–206	CCACATCTGGGTCACTCCTGTA	278–257	yes	1.90
Ptges3	TGGCTCAGTGTGGACTTCAATAA	623–645	GTGATCCATCATCTCAGAGAAACG	718–695	yes	1.90
Ptgs1	CCAGGAGCTCACAGGAGAGAA	1577–1597	ACCCCGGGTAGAACTCTAAAGC	1662–1641	yes	1.94
Rbbp9	TGGCCTTGGACATTAAGTTGAA	1157–1178	CTGGCATCCGTGTAGGACACT	1241–1221	yes	1.92
Rnase2a	CCACAAAGCAGACTGGGAAAC	25–45	TGAGGCAAGCATTAGGACATGT	117–96	yes	1.81
Sfrp5	TGTGCCCAGTGTGAGATGGA	652–671	GCGCATCTTGACCACAAAGTC	732–712	yes	1.61
Slco2a1	CCTGTATCCGGTGGAACTACCT	1866–1887	AGCAGTGTGCCCAAGACCTT	1989–1970	yes	1.82
Spi16	TGACCGCCCATTCCTTTTC	363–381	GAAGAGAACCTGCCACAGAACAA	434–412	no	1.95
Spink8	GGCCAGCTCAGTGTGGACTT	171–190	AAGCTCCCCGGTCATGTG	243–226	yes	1.98

## Data Availability

The datasets generated and analyzed during the current study are available from the corresponding author on reasonable request, in compliance with institutional policies of Pennington Biomedical Research Center/Louisiana State University System.

## Supplementary Materials

The following are available online at https://www.mdpi.com/article/10.3390/genes13010130/s1, File S1: Manufacturer’s datasheets for composition of LabDiet Purina 5001 and LabDiet Purina 5015, as downloaded from https://www.labdiet.com/Products/StandardDiets/index.html accessed on 31 October 2021.
