# Supplementary material for: Effects of Maternal Diabetes and Diet on Gene Expression in the Murine Placenta
Source: Genes (Basel). 2022 Jan 12;13(1):130. doi: 10.3390/genes13010130 (PMC8775503; doi:10.3390/genes13010130)
Supplement: Supplementary file 1 [file genes-13-00130-s001.zip › File S1.pdf]

Laboratory Rodent Diet 5001*

DESCRIPTION

Laboratory Rodent Diet is recommended for rats, mice, hamsters and gerbils. This diet is a complete life cycle diet formulated using managed formulation, delivering Constant Nutrition®. This is paired with the selection of highest quality ingredients to assure minimal inherent biological variation in long-term studies. It is formulated for life-cycle nutrition; however, it is not designed for maximizing production in mouse breeding colonies. This product has been the standard of biomedical research for over 70 years.

Features and Benefits

- Managed Formulation delivers Constant Nutrition®
- High quality animal protein added to create a superior balance of amino acids for optimum performance
- Formulated for multiple species for single product inventory
- The rodent diet standard for biomedical research

Product Forms Available	Catalog #
• Oval pellet, 3/8" x 5/8" x 1", 50 lb	0001319
• Oval pellet, 3/8" x 5/8" x 1", 15 kg	0006505
• Meal (ground pellets), 15 kg	0006508*
• Meal (ground pellets), 50 lb	0001320

* For ordering, contact info@LabDiet.com

Other Irradiated Versions Available	Catalog #
• 5L0D PicoLab® Laboratory Rodent Diet, Pelleted, Irradiated, 30 lb	3005659-220
• 5L0D PicoLab® Laboratory Rodent Diet, Meal (ground pellets), Irradiated, 30 lb	3005659-020

GUARANTEED ANALYSIS

Crude protein not less than	23.00%
Crude fat not less than	4.50%
Crude fiber not more than	6.00%
Moisture not more than	12.00%
Ash not more than	8.00%

INGREDIENTS

Ground Corn, Dehulled Soybean Meal, Dried Plain Beet Pulp, Fish Meal, Ground Oats, Dehydrated Alfalfa Meal, Brewers Dried Yeast, Cane Molasses, Wheat Germ, Dried Whey, Porcine Animal Fat Preserved with BHA and Citric Acid, Porcine Meat and Bone Meal, Wheat Middlings, Salt, Calcium Carbonate, DL-Methionine, Choline Chloride, Cholecalciferol (Vitamin D3), Vitamin A Acetate, Folic Acid, Menadione Dimethylpyrimidinol Bisulfite (Vitamin K), Pyridoxine Hydrochloride, Thiamine Mononitrate, Nicotinic Acid, Calcium Pantothenate, DL-Alpha Tocopheryl Acetate (Vitamin E), Manganous Oxide, Vitamin B12 Supplement, Zinc Oxide, Ferrous Carbonate, Copper Sulfate, Ferrous Sulfate, Riboflavin Supplement, Zinc Sulfate, Calcium Iodate, Cobalt Carbonate, Biotin, Sodium Selenite.

FEEDING DIRECTIONS

Feed ad libitum to rodents. Plenty of fresh, clean water should be available to the animals at all times.

Rats- All rats will eat varying amounts of feed depending on their genetic origin. Larger strains will eat up to 30 grams per day. Smaller strains will eat up to 15 grams per day. Feeders in rat cages should be designed to hold two to three days supply of feed at one time.

Mice-Adult mice will eat up to 5 grams of pelleted ration daily. Some of the larger strains may eat as much as 8 grams per day per animal. Feed should be available on a free choice basis in wire feeders above the floor of the cage.

Hamsters-Adults will eat up to 14 grams per day.

For information regarding shelf life please visit www.labdiet.com.

CHEMICAL COMPOSITION[1]

Nutrients[2]

**Protein, %**	**24.1**
Arginine, %	1.51
Cystine, %	0.38
Glycine, %	1.24
Histidine, %	0.60
Isoleucine, %	1.03
Leucine, %	1.83
Lysine, %	1.43
Methionine, %	0.60
Phenylalanine, %	1.06
Tyrosine, %	0.74
Threonine, %	0.94
Tryptophan, %	0.27
Valine, %	1.12
Serine, %	1.13
Aspartic Acid, %	2.71
Glutamic Acid, %	4.54
Alanine, %	1.42
Proline, %	1.43
Taurine, %	0.03
**Fat (ether extract), %**	**5.1**
**Fat (acid hydrolysis), %**	**6.4**
Cholesterol, ppm	196
Linoleic Acid, %	1.25
Linolenic Acid, %	0.12
Arachidonic Acid, %	0.02
Omega-3 Fatty Acids, %	0.31
Total Saturated Fatty Acids, %	1.39
Total Monounsaturated Fatty Acids, %	1.52
**Fiber (Crude), %**	**5.3**
Neutral Detergent Fiber[3], %	16.9
Acid Detergent Fiber[4], %	7.0
**Nitrogen-Free Extract (by difference), %**	**48.1**
Starch, %	21.9
Sucrose, %	3.25
**Total Digestible Nutrients,%**	**73.0**
**Gross Energy, kcal/gm**	**4.08**
**Physiological Fuel Value[5], kcal/gm**	**3.35**
**Metabolizable Energy, kcal/gm**	**2.86**

Minerals

**Ash, %**	**7.2**
Calcium, %	0.95
Phosphorus, %	0.67
Phosphorus (non-phytate), %	0.41
Potassium, %	1.22
Magnesium, %	0.21
Sulfur, %	0.33
Sodium, %	0.39
Chloride, %	0.65
Fluorine, ppm	15
Iron, ppm	240
Zinc, ppm	75
Manganese, ppm	71
Copper, ppm	13
Cobalt, ppm	0.96
Iodine, ppm	1.00
Chromium (added), ppm	0.01
Selenium, ppm	0.41

Vitamins

Carotene, ppm	2.5
Vitamin K, ppm	1.3
Thiamin, ppm	16
Riboflavin, ppm	4.7
Niacin, ppm	130
Pantothenic Acid, ppm	24
Choline, ppm	1840
Folic Acid, ppm	7.2
Pyridoxine, ppm	6.1
Biotin, ppm	0.30
B_{12}, mcg/kg	51
Vitamin A, IU/gm	18
Vitamin D_3 (added), IU/gm	4.6
Vitamin E, IU/kg	42
Ascorbic Acid, mg/gm	0.0

Calories provided by:

Protein, %	28.903
Fat (ether extract), %	13.606
Carbohydrates, %	57.491

1.Formulation based on calculated values from the latest ingredient analysis information. Since nutrient composition of natural ingredients varies and some nutrient loss will occur due to manufacturing processes, analysis will differ accordingly.

2. Nutrients expressed as percent of ration except where otherwise indicated. Moisture content is assumed to be 10.0% for the purpose of calculations.

3. NDF = approximately cellulose, hemi-cellulose and lignin.

4. ADF = approximately cellulose and lignin.

5. Physiological Fuel Value (kcal/gm) = Sum of decimal fractions of protein, fat and carbohydrate (use Nitrogen Free Extract) x 4,9,4 kcal/gm respectively.

NOTE: When assayed, actual levels may vary from calculated values.

LabDiet®
www.labdiet.com

Mouse Diet 5015

DESCRIPTION

Mouse Diet is high energy diet specifically designed to support reproduction, growth and maintenance of mice. This diet is a complete life cycle diet formulated using managed formulation, delivering Constant Nutrition®. This is paired with the selection of highest quality ingredients to assure minimal inherent biological variation in long-term studies. It contains 11% fat to fulfill the metabolic needs of certain mouse strains. Mouse Diet is beneficial in maintaining maximum reproduction for postpartum matings where females are under simultaneous stress of lactation and gestation.

Features and Benefits

- Managed Formulation delivers Constant Nutrition®
- A high-energy diet formulated specifically for all mouse colonies
- Helps maintain maximum reproduction for postpartum matings
- Recommended for mice with low feed intake to improve performance

Product Forms Available	Catalog #
• Oval pellet, 3/8" x 5/8" x 1", 50 lb	0001328
• Meal (ground pellets)	**0005309
**Other Irradiated Versions Available**	**Catalog #**
• 5LJ5: PicoLab® High Energy Mouse Diet, Irradiated, 30 lb	3005992-220
• 5LP1: Pico-Vac® High Energy Mouse Diet, Irradiated 5 lb vacuum sealed, 6 per box (30 lb box)	0055212

* For ordering, contact info@LabDiet.com

GUARANTEED ANALYSIS

Crude protein not less than	17.00%
Crude fat not less than	11.00%
Crude fiber not more than	3.00%
Moisture not more than	12.00%
Ash not more than	6.50%

INGREDIENTS

Ground Wheat, Dehulled Soybean Meal, Ground Corn, Wheat Germ, Brewers Dried Yeast, Porcine Animal Fat Preserved with BHA and BHT and Citric Acid, Condensed Whey, Condensed Whey Solubles, Calcium Carbonate, Soybean Oil, Dried Whey Protein Concentrate, Salt, Mono and Diglycerides of Edible Fats, DL-Methionine, Dicalcium Phosphate, Menadione Dimethylpyrimidinol Bisulfite (Vitamin K), Choline Chloride, Pyridoxine Hydrochloride, Cholecalciferol (Vitamin D3), Vitamin A Acetate, Manganous Oxide, DL-Alpha Tocopheryl Acetate (Vitamin E), Zinc Oxide, Folic Acid, Ferrous Carbonate, Vitamin B12 Supplement, Ferrous Sulfate, Thiamine Mononitrate, Calcium Pantothenate, Copper Sulfate, Nicotinic Acid, Riboflavin Supplement, Zinc Sulfate, Calcium Iodate, Cobalt Carbonate, Sodium Selenite, Biotin.

FEEDING DIRECTIONS

Mouse Diet should be fed to breeders and lactating females on a free-choice basis. Plenty of fresh, clean water should be available to the animals at all times.

Mice-Adult mice will eat up to 5 grams of pelleted ration daily. Some of the larger strains may eat as much as 8 grams per day per animal. Feed should be available on a free choice basis in wire feeders above the floor of the cage.

For information regarding shelf life please visit www.labdiet.com.

CHEMICAL COMPOSITION[1]

Nutrients[2]

**Protein, %**	**19.0**
Arginine, %	1.16
Cystine, %	0.37
Glycine, %	0.81
Histidine, %	0.47
Isoleucine, %	0.85
Leucine, %	1.43
Lysine, %	1.05
Methionine, %	0.61
Phenylalanine, %	0.87
Tyrosine, %	0.55
Threonine, %	0.72
Tryptophan, %	0.24
Valine, %	0.90
Serine, %	1.01
Aspartic Acid, %	2.04
Glutamic Acid, %	4.18
Alanine, %	1.00
Proline, %	1.30
Taurine, %	0.00
**Fat (ether extract), %**	**11.1**
**Fat (acid hydrolysis), %**	**12.0**
Cholesterol, ppm	31
Linoleic Acid, %	2.08
Linolenic Acid, %	0.16
Arachidonic Acid, %	0.03
Omega-3 Fatty Acids, %	0.21
Total Saturated Fatty Acids, %	3.70
Total Monounsaturated Fatty Acids, %	3.95
**Fiber (Crude), %**	**2.3**
Neutral Detergent Fiber[3], %	10.1
Acid Detergent Fiber[4], %	2.9
**Nitrogen-Free Extract (by difference), %**	**51.7**
Starch, %	33.5
Sucrose, %	0.90
**Total Digestible Nutrients,%**	**85.2**
**Gross Energy, kcal/gm**	**4.74**
**Physiological Fuel Value[5], kcal/gm**	**3.83**
**Metabolizable Energy, kcal/gm**	**3.58**

Minerals

**Ash, %**	**5.8**
Calcium, %	0.80
Phosphorus, %	0.50
Phosphorus (non-phytate), %	0.25
Potassium, %	0.80
Magnesium, %	0.15
Sulfur, %	0.25
Sodium, %	0.43
Chloride, %	0.70
Fluorine, ppm	8.4
Iron, ppm	170
Zinc, ppm	110
Manganese, ppm	120
Copper, ppm	17
Cobalt, ppm	0.63
Iodine, ppm	1.45
Chromium (added), ppm	0.02
Selenium, ppm	0.30

Vitamins

Carotene, ppm	0.2
Vitamin K, ppm	3.0
Thiamin, ppm	12.5
Riboflavin, ppm	5.5
Niacin, ppm	75
Pantothenic Acid, ppm	20
Choline, ppm	1500
Folic Acid, ppm	2.9
Pyridoxine, ppm	9.6
Biotin, ppm	0.30
B_{12}, mcg/kg	51
Vitamin A, IU/gm	18
Vitamin D_3 (added), IU/gm	3.3
Vitamin E, IU/kg	66
Ascorbic Acid, mg/gm	0.00

Calories provided by:

Protein, %	19.896
Fat (ether extract), %	26.088
Carbohydrates, %	54.016

1. Formulation based on calculated values from the latest ingredient analysis information. Since nutrient composition of natural ingredients varies and some nutrient loss will occur due to manufacturing processes, analysis will differ accordingly.
2. Nutrients expressed as percent of ration except where otherwise indicated. Moisture content is assumed to be 10.0% for the purpose of calculations.
3. NDF = approximately cellulose, hemi-cellulose and lignin.
4. ADF = approximately cellulose and lignin.
5. Physiological Fuel Value (kcal/gm) = Sum of decimal fractions of protein, fat and carbo- hydrate (use Nitrogen Free Extract) x 4,9,4 kcal/gm respectively.

NOTE: When assayed, actual levels may vary from calculated values.

LabDiet®
www.labdiet.com
